# Impulsiveness, overactivity, and poorer sustained attention improve by chronic treatment with low doses of l-amphetamine in an animal model of Attention-Deficit/Hyperactivity Disorder (ADHD)

**DOI:** 10.1186/1744-9081-7-6

**Published:** 2011-03-30

**Authors:** Terje Sagvolden

**Affiliations:** 1 Institute of Basic Medical Sciences, Department of Physiology, University of Oslo, P.O. Box 1103 Blindern, NO-0317 Oslo, Norway

## Abstract

**Background:**

ADHD is currently defined as a cognitive/behavioral developmental disorder where all clinical criteria are behavioral. Overactivity, impulsiveness, and inattentiveness are presently regarded as the main clinical symptoms. There is no biological marker, but there is considerable evidence to suggest that ADHD behavior is associated with poor dopaminergic and noradrenergic modulation of neuronal circuits that involve the frontal lobes. The best validated animal model of ADHD, the Spontaneously Hypertensive Rat (SHR), shows pronounced overactivity, impulsiveness, and deficient sustained attention. The primary objective of the present research was to investigate behavioral effects of a range of doses of chronic l-amphetamine on ADHD-like symptoms in the SHR.

**Methods:**

The present study tested the behavioral effects of 0.75 and 2.2 mg l-amphetamine base/kg i.p. in male SHRs and their controls, the Wistar Kyoto rat (WKY). ADHD-like behavior was tested with a visual discrimination task measuring overactivity, impulsiveness and inattentiveness.

**Results:**

The striking impulsiveness, overactivity, and poorer sustained attention seen during baseline conditions in the SHR were improved by chronic treatment with l-amphetamine. The dose-response curves were, however, different for the different behaviors. Most significantly, the 0.75 mg/kg dose of l-amphetamine improved sustained attention without reducing overactivity and impulsiveness. The 2.2 mg/kg dose improved sustained attention as well as reduced SHR overactivity and impulsiveness.

**Discussion:**

The effects of l-amphetamine to reduce the behavioral symptoms of ADHD in the SHR were maintained over the 14 days of daily dosing with no evidence of tolerance developing.

## Background

Attention-deficit/hyperactivity disorder (ADHD) is currently defined as a cognitive developmental disorder where all clinical criteria are behavioral [1]. Overactivity, impulsiveness, and inattentiveness are presently regarded as the main clinical symptoms.

There have been many attempts to explain the origins of ADHD symptoms. A dual-process theory [2-5] suggests that less efficient reinforcement processes and deficient extinction of previously reinforced behavior may explain behavioral changes often described as response disinhibition [6] or poor executive functioning [7].

ADHD is highly heritable and the genetic and neurobiological causes are likely to reside in brain catecholamines (for a review see [4]). Most likely, ADHD symptoms are associated with reduced post-synaptic efficacy of dopaminergic and noradrenergic modulation of neuronal circuits that involve the frontal lobes [8,9]. Imaging of striatal neuronal networks indicates reduced dopamine efficacy in ADHD [10]. Further, noradrenergic systems are involved in attention processes and prime prefrontal areas for response to sensory stimuli [11]. It is therefore not surprising that amphetamines and other catecholamine agonists have been the drugs of choice in medication of ADHD [8,9,12-14]. Although catecholamine agonists improve behavior, long-term academic performance is not improved to the same extent [14-18].

The spontaneously hypertensive rat (SHR) is the best validated animal model of ADHD. These rats show hyperactivity, impulsiveness and deficits in sustained attention [9,19-22]. The control strain is usually the Wistar Kyoto Rat (WKY) as this rat is the progenitor strain and its behavior is closely similar to that of other strains when tested in well-controlled operant tasks [20]. Drugs used in the pharmacological treatment of ADHD, usually catecholamine agonists have been shown to reduce ADHD-like behavior in this model [19,22-25].

Results from animal studies indicate that higher doses of amphetamines are required for reducing SHR overactivity and impulsiveness than those required for improving SHR sustained attention which, in children, is essential for long-term academic performance [22]. Although d-amphetamine improves SHR overactivity and impulsiveness as well as sustained attention, the behavioral effects of l-amphetamine were relatively more specific for improving sustained attention than for the other two symptoms [22].

The primary objective of the present study was to test the effects of chronic administration of 0.75 and 2.2 mg l-amphetamine base/kg i.p. to male SHRs and their controls, the Wistar Kyoto rat (WKY), in a visual discrimination task measuring overactivity, impulsiveness and inattentiveness. Lower doses were used than in a previous single-dose study [22].

## Methods

### Subjects

A total number of 91 male rats, 47 SHR and 44 WKY, participated in this study. At the start of testing following 3 days acclimatization, the rats were 5 wk old and experimentally naïve. Young rats were required, as ADHD primarily is a child and adolescent disorder. Due to health status requirements, half of the SHRs were obtained from Charles River Germany (SHR/NCrl), the other half from Charles River Italy (SHR/NCrl). The WKYs were from Charles River Germany (WKY/NCrl).

At the University of Oslo, the rats were housed individually in 41 × 25 × 25 (height) cm transparent cages and had free access to food (RM3 (E) from Special Diet Services, Witham, Essex CM8 3AD, UK). The rats had access to water at all times before the habituation session. Starting following completion of the habituation session, the rats were deprived of water for 21 hr a day; this is a moderate, but sufficient deprivation for motivating the animal. The temperature in the housing area was ~22°C. The light was on from 0700 to 1900 hours. The behavioral training took place between 1000 and 1530 hours seven days a week. The experiments were approved by the Norwegian Animal Research Authority (NARA), and were conducted in accordance with the laws and regulations controlling experimental procedures in live animals in Norway and the European Union's Directive 86/609/EEC.

### Behavioral apparatus

Sixteen Campden Instruments operant chambers were used in the study. The animal's working space in eight of the chambers was 25 × 25 × 30 (height) cm and 25 × 25 × 20 (height) cm in the other eight chambers. A fan producing a low masking noise and the 2.8-W house light were on during the entire experimental session.

During training sessions, either no, one or both retractable levers were used (below). A 2.8-W cue light was located above each lever. The rats' response consisted of pressing one of the levers with a dead weight of at least 3 g to activate a micro-switch. The reinforcers (0.01 ml tap water) were delivered by a liquid dipper located in a small recessed cubicle with a 2.8-W cue light that lit up when a reinforcer was presented. A 7 × 5 cm transparent plastic lid separated the cubicle from the rat's working space. The rat could easily open the lid with a light push with the nose or paw. Each chamber was ventilated and placed in a sound-resistant outer housing. A computer and an online system (SPIDER, Paul Fray, Ltd., UK) recorded the behavior and scheduled reinforcers (drops of water).

Before the initiation of the study, the rats were assigned a chamber (1 through 16) and time of testing (10, 12 or 14 o'clock) in a randomized and balanced way. The rat was returned to its living cage after each session and immediately given free access to water for 60 min.

### Response acquisition

The training period started with a single 30-min habituation session. During the habituation session, the lid between the working space and the reinforcement cubicle was kept open. The house light was on, but no lever was present, no cue light above any lever was lit and water was not delivered.

The habituation session was followed by two dipper training sessions, lasting 30 and 15 min, respectively. The lid was taped open, no levers were present, and the house light was on, but the cue lights above the levers were not lit. The computer delivered water on the average every 10 s independent of the rat's behavior (a variable-time schedule). Each water delivery was accompanied by the turning on of the cue light in the small recessed cubicle.

In the next two 30-min sessions, the rat was trained to open the lid to gain access to the water. The lid was not taped open, no levers were present and the lights above the levers were not activated. The house light was on. Each lid opening was followed by a presentation of a single drop of water. The cue light in the recessed cubicle was turned on when water was present.

During the subsequent three to four sessions (depending on performance), lever responding was shaped by the method of successive approximations [26]. During the initial sessions, the rats learned to press the left lever in order to receive a reinforcer immediately following every press. The cue light above the left lever was now lit the entire session. The right lever was retracted into the wall. On the final session, the right lever was activated and the left lever retracted. During this session the light above the right lever was lit the entire session and the light above the left lever was off. The house light was on during both sessions. Following this shaping procedure, the animal had acquired the appropriate lever-pressing behavior.

From now on, both levers were present. The light above the levers shifted randomly. The light stayed lit above a lever for as long as it was the correct lever. This was the discriminative stimulus showing the rat which lever it had to press in order to receive a reinforcer. A concurrent extinction schedule was present on the wrong lever. There was never any light above the extinction lever. Thus, the present task was a simultaneous visual discrimination task. The seven sessions lasted for 30 min and the reinforcers were delivered following every correct lever press. Whenever an interval had elapsed, the reinforcer was delivered immediately following the first correct response.

### Final schedule

The simultaneous visual discrimination task was used for testing effects of the drugs. An unpredictable 180-s random-interval schedule was in effect for 90 min on the correct lever (signaled by a constantly lit cue light above this lever) from session 18 on until the study was finished at session 61. Inter-reinforcer times ranged from 6 to 719 s in a randomized fashion with a skewed distribution modeled after the "Harvard golden tape" [27]. There was neither any external stimulus signaling that a reinforcer was programmed, nor any external stimulus signaling the time since the last response. A concurrent extinction schedule (never associated with any cue light) was present on the wrong lever. The house light was lit the entire session.

### Behavioral measures

Each session was divided into five 18-min segments (parts) in order to monitor intra-session changes in the behavior. For each segment, total number of presses on the correct and incorrect lever as well as number of reinforcers delivered were recorded. Time between consecutive correct responses (inter-response time, IRT) was also recorded.

The total number of lever presses is an expression of the general activity level and therefore a measure of degree of *activity*. The percent choice of the correct lever when the reinforcers are delivered infrequently is a measure of *sustained attention *[22]. The number of responses with short IRTs (< 0.67 s) is used as a measure of degree of *impulsiveness *(cannot hold back a response even when one knows it is an unnecessary one).

### Drug administration

The animals were randomly assigned to three treatment groups: 0.75 or 2.2 mg/kg l-amphetamine sulphate or vehicle (physiological saline). Each rat was injected intraperitoneally at a dose volume of 1 ml/kg body weight of the animal ~30 min before testing. The daily administration of the drug started at session 45 when the behavior had stabilized and ended at session 58 except for the saline groups that received 2.2 mg/kg l-amphetamine ~30 min before sessions 59-61 in order to check that the drug response of these groups was the same as their counterparts.

### Drugs

l-Amphetamine sulphate (Lot FB-101-57) was supplied from Boeringer-Ingelheim US. Doses were calculated as the weight of base using a conversion factor of 1.360 mg sulphate salt as equivalent to 1.000 mg base. Doses were based on previously published data [22]. Dosing solutions were prepared in physiological saline. The drug solutions were prepared each day of dosing.

### Data management and statistical procedures

The mean behavior was regarded as the drug response. The data were processed by univariate and multivariate analyses of variance (ANOVAs and MANOVAs, respectively) with the Statistica 7.1 program [28]. Strain and dose were between-subject variables coded as subgroups. One control rat fell ill early in the study and had to be sacrificed. Post-hoc comparisons following MANOVAs were performed by the Newman-Keuls test.

## Results

### General

**Figure 1:**
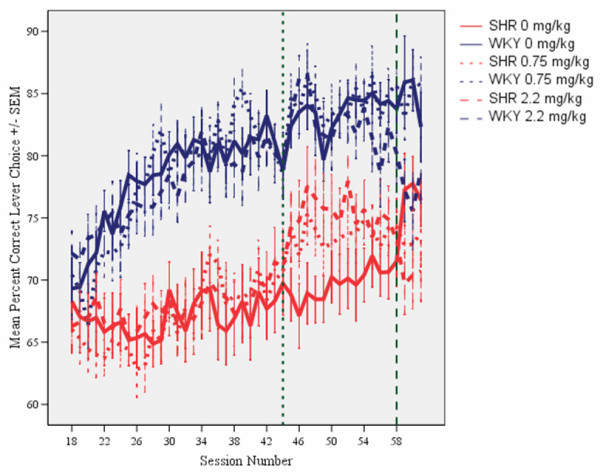
**Effects of l-amphetamine on sustained attention, choice of the correct lever in percent of all lever presses, by SHR and WKY controls**. Means ± SEM. Sessions 31 through 44 served as baseline sessions. Treatment was given at sessions 45 through 58. Sessions 59 through 61 were used for studying post-treatment effects, except for the groups that received vehicle (saline, 0 mg/kg) during treatment sessions 45-58. These groups received 2.2 mg/kg l-amphetamine to check for typical responsiveness to the drug for SHR and WKY.

**Figure 2:**
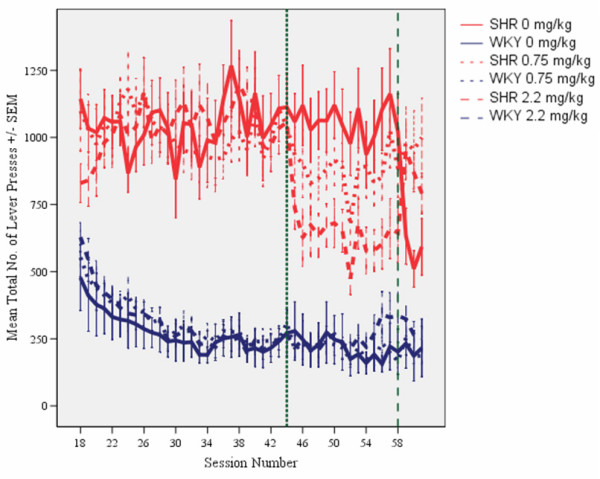
**Effects of l-amphetamine on total number of lever presses (correct plus incorrect) by SHR and WKY controls**. Means ± SEM. Sessions 31 through 44 served as baseline sessions. Treatment was given at sessions 45 through 58. Sessions 59 through 61 were used for studying post-treatment effects, except for the groups that received vehicle (saline, 0 mg/kg) during treatment sessions 45-58. These groups received 2.2 mg/kg l-amphetamine to check for typical responsiveness to the drug for SHR and WKY.

**Figure 3:**
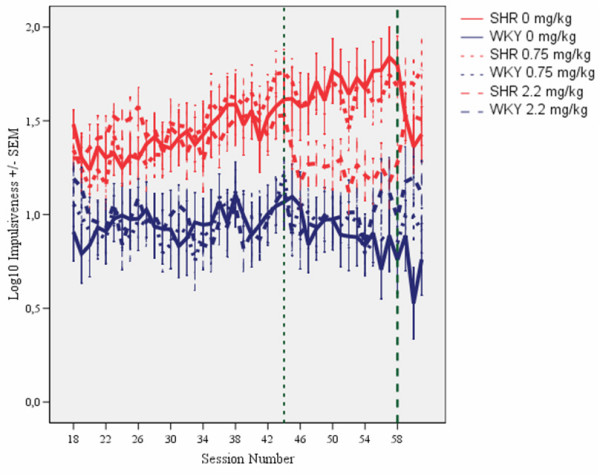
**Effects of l-amphetamine on impulsiveness, responding within 0.67 s following the previous lever press, of SHR and WKY controls following log10 transformation**. Means ± SEM. Sessions 31 through 44 served as baseline sessions. Treatment was given at sessions 45 through 58. Sessions 59 through 61 were used for studying post-treatment effects, except for the groups that received vehicle (saline, 0 mg/kg) during treatment sessions 45-58. These groups received 2.2 mg/kg l-amphetamine to check for typical responsiveness to the drug for SHR and WKY.

Compared to WKY controls, SHRs showed poorer sustained attention (Figure 1), pronounced overactivity (Figure 2), and impulsiveness (Figure 3) (see Additional files 1 and 2). There were clear dose-response curves to l-amphetamine in the SHR, but the dose-response curves were different for the different behaviors. The 0.75 mg/kg dose improved SHR sustained attention, without reducing overactivity and impulsiveness. The 2.2 mg/kg dose improved attention, overactivity and impulsiveness in the SHR. All of these effects were maintained over the 14 days of daily dosing. The effects were only present during sessions with active drug. The drug had little effect on WKY behavior.

### Acquisition

As is the case in children with ADHD [29,30], the symptoms in the SHR developed with time, but differently for the different behaviors [21]. The final schedule was installed on session 18. Sustained attention improved in the WKY controls and stabilized at about session 30 (Figure 1). A pronounced overactivity was seen in SHRs from session 18 onwards (Figure 2). SHR impulsiveness, responding within 0.67 s since the previous lever press although such a lever press was rarely reinforced, continued to increase in the SHR throughout the entire study [21]. This measure was accompanied by increased variability over days during the course of the study, something that is typical in ADHD [31-33]. Impulsiveness was subjected to a log10-transformation in order to obtain the more equal variances required by the ANOVAs (Figure 3). For all three behaviors, the three SHR subgroups were closely similar before the start of the injection program. So were the three WKY subgroups.

### Sustained attention

Without medication, SHRs showed poorer sustained attention than WKY controls. The three SHR subgroups were closely similar before the start of the injection program, as were the three WKY subgroups (Figure 1).

**Figure 4:**
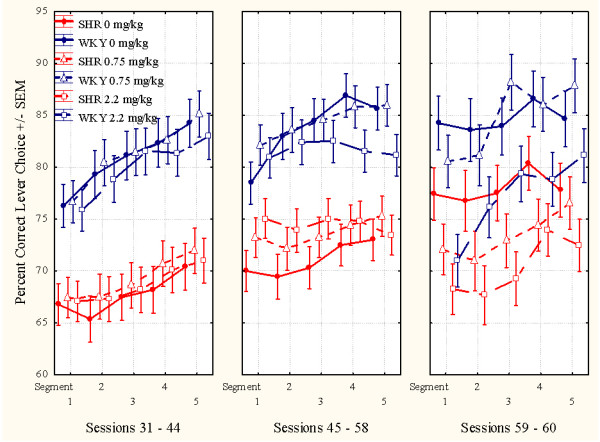
**This figure shows the mean within-session effects of l-amphetamine on sustained attention**. **Left panel**: Sessions 31 through 44, baseline sessions. The three SHR subgroups are closely similar, so are the three WKY subgroups, prior to the injection program. **Middle panel**: Sessions 45 through 58, treatment sessions. Both doses improve SHR behavior. **Right panel**: Sessions 59 through 61, post-treatment sessions. The SHR subgroups return to pre-drug levels. The 2.2 mg/kg WKY subgroup apparently got worse following drug exposure. The WKY performance appears unaltered from pre- to post-treatment days. The groups that had received saline during sessions 45 through 58, now received 2.2 mg/kg.

L-amphetamine produced a dose-related improvement in sustained attention in the SHR, but not in the WKY. This improvement was maintained throughout the 14 day dosing period (sessions 45-58) (Figure 1). A similar effect of the 2.2 mg/kg dose was seen in the SHR subgroup which received vehicle (saline, 0 mg/kg) during sessions 45 through 58 and then received the drug during sessions 59 through 61 (Figures 1 and 4). The drug effect did not transfer to sessions following the cessation of drug administration. For the WKYs, 0.75 mg/kg l-amphetamine did not alter attentional behavior, but the 2.2 mg/kg dose produced a slight deterioration in behavior after dosing was terminated (Figure 4).

#### Acute and chronic effects of l-amphetamine

The ANOVA comparing effects during pre-drug treatment (days 31 to 44) with effects during drug treatment (days 45 to 58) showed a statistically significant main effect of subgroup (*F(3,87) = 14.62, p < 0.001*). The MANOVA showed a main effect of treatment (*F(1,87) = 52.21, p < 0.001)*, a main effect of segment of session (*F(4,84) = 12.98, p < 0.001)*, a 2-way subgroup × treatment interaction (*F(3,87) = 6.63, p < 0.001*), but no 3-way subgroup × treatment × segment interaction (*F(12,223) = 1.36, p > 0.1*). The acute drug effects in the subgroups receiving 2.2 mg/kg during sessions 59 through 61 were compared to their behavior during sessions 45 through 58, when they received saline, and combined with the other animals of the same strain receiving 2.2 mg/kg during sessions 45 through 58. Thus, the drug had a larger effect in SHR than in WKY controls (Figures 1 and 4).

In order to check for the stability of the drug effects over the 14 days of daily dosing, the 14 treatment sessions were divided into two halves, the initial seven sessions and the final seven sessions. The MANOVA of the sustained attention behavior of the subgroups receiving an active dose showed no statistically significant development from the 1^st ^to the 2^nd ^half of the test period. The subgroup × half test period interaction was: *F(3,58) = 0.96, p > 0.4*).

### Overactivity

**Figure 5:**
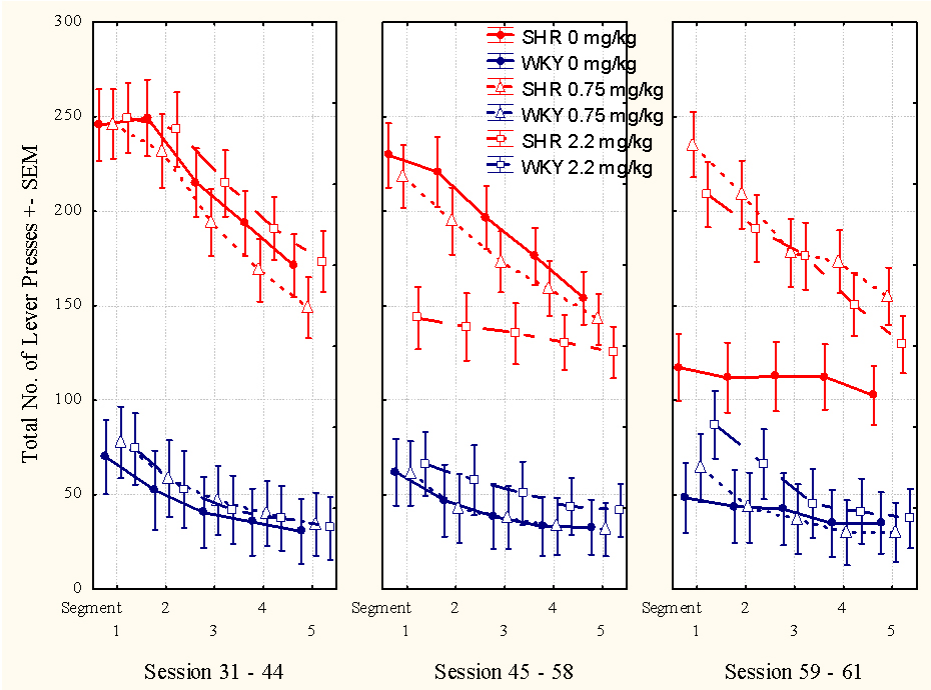
**This figure shows the mean within-session effects of l-amphetamine on activity**. **Left panel**: Sessions 31 through 44, baseline sessions. The three SHR subgroups are closely similar, so are the three WKY subgroups, prior to the injection program. **Middle panel**: Sessions 45 through 58, treatment sessions. 2.2 mg/kg reduces SHR overactivity. **Right panel**: Sessions 59 through 61, post-treatment sessions. The 2.2 mg/kg SHR subgroup returns to pre-drug levels. The WKY subgroups are apparently unaffected by the drug. The subgroups that had received saline during sessions 45 through 58, now received 2.2 mg/kg.

Without medication, SHRs showed a substantially higher activity than WKY controls. The three SHR subgroups were closely similar before the start of the injection program, as were the three WKY subgroups (Figures 2 and 5). L-amphetamine, 2.2 mg/kg, reduced hyperactivity in the SHR, whilst having no effect on activity in the WKY. The improvement in the SHR was maintained throughout the 14-day dosing period (Figure 2). A similar effect of the 2.2 mg/kg dose was seen in the SHR subgroup which received vehicle (saline, 0 mg/kg) during sessions 45 through 58 and then received the drug during sessions 59 through 61 (Figures 2 and 5). The 2.2 mg/kg drug effect was not transferred to sessions following the cessation of drug administration. The 0.75 mg/kg dose had no effect. There was no apparent effect of the drug in the WKY controls.

#### Acute and chronic effects of l-amphetamine

The ANOVA showed a statistically significant main effect of subgroup (*F(3,87) = 34.10, p < 0.001*). The MANOVA showed a main effect of treatment (*F(1,87) = 52.81, p < 0.001)*, a main effect of segment of session (*F(4,84) = 62.23, p < 0.001)*, a 2-way subgroup × treatment interaction (*F(3,87) = 34.20, p < 0.001*), a 2-way subgroup × segment of session interaction (*F(12,223) = 6.35, p < 0.001*), and a 3-way subgroup × treatment × segment interaction (*F(12,223) = 5.91, p < 0.001*). The acute drug effects in the subgroups receiving 2.2 mg/kg during sessions 59 through 61 were compared to their behavior during sessions 45 through 58, when they received saline, and combined with the other animals of the same strain receiving 2.2 mg/kg during sessions 45 through 58. Thus, the results showed that the SHR receiving 2.2 mg/kg had a larger effect in SHR than in WKY controls (Figures 2 and 5).

Stability of the drug effects over the 14 days of daily dosing was checked by dividing the 14 treatment sessions, into two halves, the initial seven sessions and the final seven sessions. The MANOVA of the activity of the subgroups receiving an active dose showed no statistically significant development from the 1^st ^to the 2^nd ^half of the test period. The subgroup × half test period interaction was: *F(3,58) = 1.70, p > 0.15*.

### Impulsiveness

**Figure 6:**
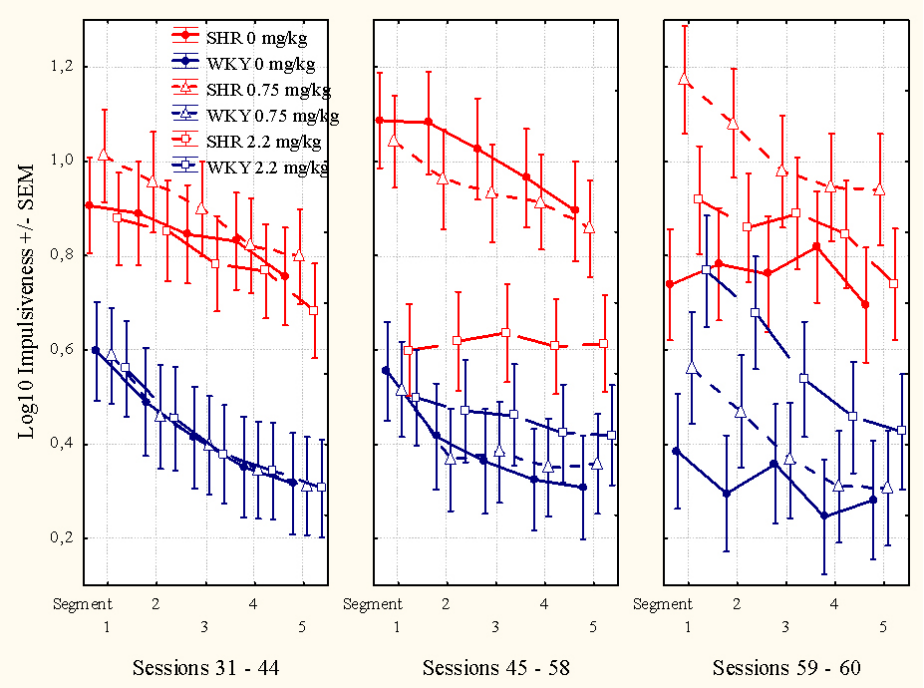
**This figure shows the mean within-session effects of l-amphetamine**. **Left panel**: Sessions 31 through 44, baseline sessions. The three SHR subgroups are closely similar, so are the three WKY subgroups, prior to the injection program. **Middle panel**: Sessions 45 through 58, treatment sessions. The 2.2 mg/kg dose reduced SHR impulsiveness. **Right panel**: Sessions 59 through 61, post-treatment sessions. The 2.2 mg/kg SHR subgroup returned to pre-drug levels. The 2.2 mg/kg WKY subgroup apparently got worse following drug exposure. The subgroups that had received saline during sessions 45 through 58, now received 2.2 mg/kg l-amphetamine.

Without medication, SHRs were substantially more impulsive than WKY controls. The three SHR subgroups were closely similar before the start of the injection program, as were the three WKY subgroups (Figures 3 and 6). L-amphetamine, 2.2 mg/kg, reduced impulsiveness in the SHR, whilst having no effect in the WKY. The improvement in impulsiveness was maintained throughout the 14-day dosing period (Figure 3). A similar effect of the 2.2 mg/kg dose was seen in the SHR subgroup which received vehicle (saline, 0 mg/kg) during sessions 45 through 58 and then received the drug during sessions 59 through 61 (Figure 3). The 0.75 mg/kg dose had no effect in any of the subgroups. The 2.2 mg/kg drug effect was not transferred to sessions following the cessation of drug administration.

#### Acute and chronic effects of l-amphetamine

The ANOVA showed a statistically significant main effect of subgroup (*F(3,87) = 10.00, p < 0.001*). The MANOVA did not show a main effect of treatment (*F(1,87) = 2.96, p > 0.08)*, but a main effect of segment of session (*F(4,84) = 32.31, p < 0.001)*, a 2-way subgroup × treatment interaction (*F(3,87) = 3.77, p < 0.02*), a 2-way subgroup × segment of session interaction (*F(12,223) = 2.73, p < 0.002*), but no 3-way subgroup × treatment × segment interaction (*F(12,223) = 0.94, p > 0.5*). The acute drug effects in the subgroups receiving 2.2 mg/kg during sessions 59 through 61 were compared to their behavior during sessions 45 through 58, when they received saline, and combined with the other animals of the same strain receiving 2.2 mg/kg during sessions 45 through 58. Thus, the results showed that the SHR receiving 2.2 mg/kg had a larger effect in SHR than in WKY controls (Figures 3 and 6).

Stability of the drug effects over the 14 days of daily dosing was checked by dividing the 14 treatment sessions into two halves, the initial seven sessions and the final seven sessions. The MANOVA of the impulsiveness of the subgroups receiving an active dose no statistically significant development from the 1^st ^to the 2^nd ^half of the test period. The subgroup × half test period interaction was: *F(3,58) = 1.27, p > 0.4*.

### Reinforcers delivered

The random-interval reinforcement schedule used was programmed so that even large individual differences in lever pressing would result in approximately 6 reinforcers (drops of water) during each 18-min segment of the session, even for the case of the less active group. A major advantage of such a schedule is the fact that systematic strain differences in thirst should not be of concern when interpreting the data. The results show that both strains in general also received 6 reinforcers per segment during active drug.

### Stereotypy and severely drugged behavior

These doses of l-amphetamine did not produce stereotypy or severely drugged behavior in the animals.

## Discussion

ADHD is currently defined as a cognitive/behavioral developmental disorder where all clinical criteria are behavioral. Overactivity, impulsiveness, and inattentiveness are presently regarded as the main clinical symptoms [1]. These symptoms have been operationalized in a long series of translational research studies investigating ADHD behavior in children and animal models (e.g. [30-35]).

ADHD is highly heritable and the genetic and neurobiological causes are likely to reside in reduced postsynaptic effects of catecholamines on glutamatergic and GABAergic neurons [4]. These changes apparently cause less efficient reinforcement processes and deficient extinction of previously reinforced behavior [3-5].

Amphetamines and other dopamine agonists have been the drugs of choice in medication of ADHD [8,9,12-14]. The primary objectives of the present research were to test the behavioral effects of chronic administration of 0.75 and 2.2 mg/kg l-amphetamine in SHRs and their controls, the Wistar Kyoto rat (WKY) in a visual discrimination task measuring overactivity, impulsiveness and inattentiveness.

The results showed that both doses of l-amphetamine improved sustained attention in the SHR, but only the 2.2 mg/kg dose reduced SHR overactivity and impulsiveness. These effects are similar to those seen previously after acute administration of the drug [22]. The current study has shown that these acute effects of l-amphetamine are maintained with repeated daily dosing over 14 days, with no evidence of tolerance developing. Results from the present as well as a previous study [22] indicate that higher doses of amphetamines are required for reducing SHR overactivity and impulsiveness than those required for improving SHR sustained attention.

In conclusion, low doses of l-amphetamine improved sustained attention while higher doses improved sustained attention as well as overactivity and impulsiveness in the SHR. These effects were maintained on chronic dosing.

## Competing interests

This research was in part financially supported by Shire Pharmaceutical Development LTD, England (Company No. 2486738), Hampshire International Business Park, Chineham, Basingstoke, Hampshire RG24 8EP, Great Britain. The company had no role, however, in the presentation of the research. Data presentation, statistics, discussion and conclusions that are the author's own responsibility.

## Supplementary Material

Additional file 1 The video shows a normal male WKY control rat performing the visual discrimination task. Click here for file

Additional file 2 The video shows a Spontaneously Hypertensive Rat (SHR) performing the visual discrimination task. The rat is overactive and inattentive. Click here for file
